# ApoE4 (Δ272–299) induces mitochondrial‐associated membrane formation and mitochondrial impairment by enhancing GRP75-modulated mitochondrial calcium overload in neuron

**DOI:** 10.1186/s13578-021-00563-y

**Published:** 2021-03-06

**Authors:** Tao Liang, Weijian Hang, Jiehui Chen, Yue Wu, Bin Wen, Kai Xu, Bingbing Ding, Juan Chen

**Affiliations:** 1 Department of Biochemistry and Molecular Biology, School of Basic Medicine and the Collaborative Innovation Center for Brain Science, Tongji Medical College, Huazhong University of Science and Technology, 430030 Wuhan, China; 2 Department of Clinical laboratory, Union Hospital, Tongji Medical College, Huazhong University of Science and Technology, 430022 Wuhan, China; 3 Division of Cardiology, Department of Internal Medicine, Tongji Hospital, Tongji Medical College, Huazhong University of Science and Technology, 430030 Wuhan, China

**Keywords:** Alzheimer’s disease, Apolipoprotein E4, apoE4 (Δ272–299), ER stress, Mitochondria‐associated ER membrane, Mitochondrial Ca^2+^ overload

## Abstract

**Background:**

Apolipoprotein E4 (apoE4) is a major genetic risk factor of Alzheimer’s disease. Its C-terminal-truncated apoE4 (Δ272–299) has neurotoxicity by affecting mitochondrial respiratory function. However, the molecular mechanism(s) underlying the action of apoE4 (Δ272–299) in mitochondrial function remain poorly understood.

**Methods:**

The impact of neuronal apoE4 (Δ272–299) expression on ER stress, mitochondrial-associated membrane (MAM) formation, GRP75, calcium transport and mitochondrial impairment was determined *in vivo* and *in vitro*. Furthermore, the importance of ER stress or GRP75 activity in the apoE4 (Δ272–299)-promoted mitochondrial dysfunction in neuron was investigated.

**Results:**

Neuronal apoE4 (Δ272–299) expression induced mitochondrial impairment by inducing ER stress and mitochondrial-associated membrane (MAM) formation *in vivo* and *in vitro*. Furthermore, apoE4 (Δ272–299) expression promoted GRP75 expression, mitochondrial dysfunction and calcium transport into the mitochondria in neuron, which were significantly mitigated by treatment with PBA (an inhibitor of ER stress), MKT077 (a specific GRP75 inhibitor) or GRP75 silencing.

**Conclusions:**

ApoE4 (Δ272–299) significantly impaired neuron mitochondrial function by triggering ER stress, up-regulating GRP75 expression to increase MAM formation, and mitochondrial calcium overload. Our findings may provide new insights into the neurotoxicity of apoE4 (Δ272–299) against mitochondrial function and uncover new therapeutic targets for the intervention of Alzheimer’s disease.
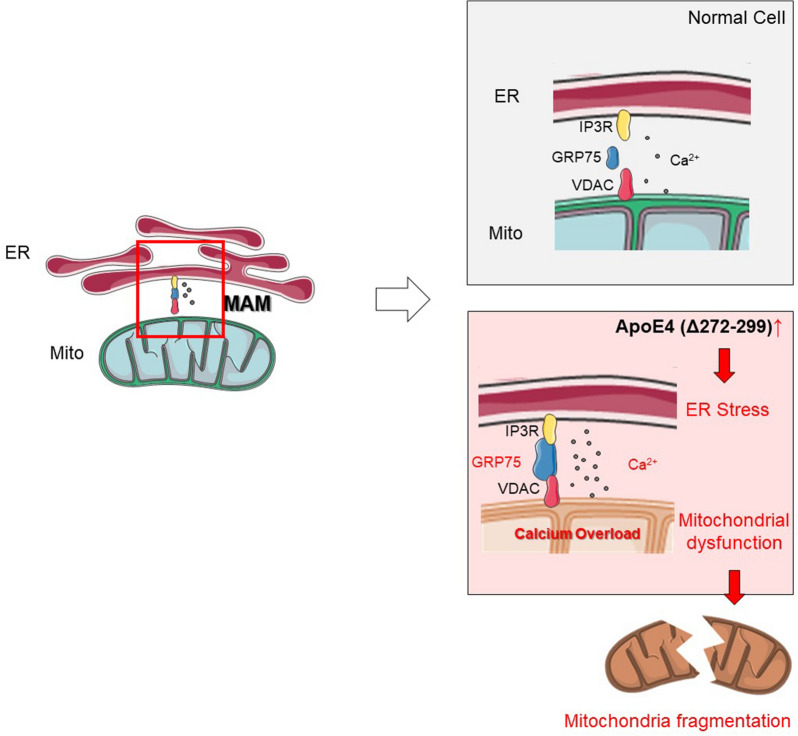

## Introduction

Alzheimer’s disease (AD) is a common neurodegenerative disease and is caused by several pathogenic factors, including the genetic susceptibility [1, 2]. It is well known that one of the most important genetic risk factors for AD is apolipoprotein E4 (apoE4). Approximately, 40–65 % of patients with familial and sporadic AD carry the apoE4 allele. While individuals carrying apoE ε4 homozygotes have a 12-fold increased risk for AD, those carrying apoE ε4 heterozygotes have a 2- to 3-fold increased risk for AD and the apoE4 reduces the age of AD onset [3]. Previous studies have shown that the neuron-specific expression of apoE4 can cause AD-like pathological changes in the brain and learning memory impairments in transgenic mice [4, 5]. It is notable that the apoE4 can be cleaved at Met 272 to produce apoE4 (Δ272–299) fragment, which has been detected in the brain of apoE4 transgenic mice and AD patients [6]. Furthermore, the levels of apoE4 (Δ272–299) in the transgenic mouse brain were closely related to the degrees of pathological manifestation, including learning memory dysfunction and neuronal degeneration [7]. Therefore, we speculate that apoE4 (Δ272–299) may be an effector molecule of apoE4 for the neuronal pathogenesis of AD.

The disorders of mitochondrial morphology and function occur in the early stage of AD, even before the formation of Aβ plaque and Tau hyperphosphorylation tangles [8–10]. However, how the apoE4 plays in mitochondrial dysfunction and the pathogenesis of AD is still not fully understood. Previous studies have found that mitochondrial dysfunction is an earlier event than the occurrence of AD pathological changes in AD patients carrying the apoE4 gene and apoE4 transgenic mice [11, 12]. In addition, neuronal damage or stress can enhance apoE4 expression and cleavage into apoE4 (Δ272–299) in rodents [6]. ApoE4 (Δ272–299) over-expression can damage the mitochondrial respiratory chain-related complex in neurons, which is more effective than apoE4 13]. However, it is still unclear whether and how the apoE4 (Δ272–299) affects neuronal mitochondrial morphology and function.

The mitochondria-associated ER membrane (MAM) connects the endoplasmic reticulum (ER) with mitochondria, and is crucial for exchanging information and transferring substances between the ER and mitochondria. Currently, the MAM is important for cholesterol synthesis, lipid metabolism, calcium homeostasis, oxidative stress, mitochondria quality control and others [14–17]. The disturbance of MAM structure is associated with the development of various diseases, including AD [18–22]. The MAM is crucial for the transport of Ca^2+^ between the mitochondria and ER to maintain mitochondrial morphology and function. The alternation in Ca^2+^ transport and subsequent mitochondrial Ca^2+^ overload contributes to the pathogenesis of AD [23, 24]. Actually, the changes in MAM structure can interfere with calcium signaling and calcium homeostasis in AD 25].

It is well-known that the IP3R-GRP75-VDAC complex is a canonical pathway to mediate Ca^2+^ transport between the ER and mitochondria through the MAM. Glucose-regulated protein 75 (GRP75) functions to link the IP3R to VDAC in the complex, and maintains the conformational stability of IP3R [26]. Down-regulated GRP75 expression is associated with a decrease in mitochondrial Ca^2+^ levels [27]. Accordingly, up-regulated GRP75 expression may enhance the calcium transporting function of MAM, and increase mitochondrial Ca^2+^ load.

In this study, we investigated the possible mechanisms underlying the neurotoxic effects of apoE4 (Δ272–299) on mitochondrial dysfunction. We found that neuron-specific apoE4 (Δ272–299) expression triggered ER stress, and promoted the interaction between the ER and mitochondria, calcium transport and mitochondrial Ca^2+^ overload, disrupting mitochondrial morphology and function.

## Materials and methods

### Materials

This study used monoclonal antibodies (mAb) against Mitofusin1 (Mfn1, #14,739), Mitofusin2 (Mfn2, #11,925), Optic atrophy 1 (OPA1, #67,589), Mitochondrial fission factor (MFF, #84,580), Dynamin-related protein 1 (Drp1, #8570), p-Drp1 Ser^616^ (#4494, Cell Signaling Technology, Beverly, MA, USA); Glucose-regulated protein 78 (GRP78, ab21685) and β-actin (ab8226, Abcam. Cambridge, MA, USA); polyclonal antibodies (pAb) against GRP75 (A0558), C/EBP homologous protein (CHOP, A0221, Abclonal, Wuhan, China). Other specific reagents included MitoTracker® Deep Red FM (M22426) and ER-Tracker™ Blue (E12353), bicinchoninic acid (BCA) protein detection kit (#23,227, Thermo Fisher Scientific, Waltham, MA, USA), polyvinylidene difluoride (PVDF) membrane (GE Health, New York, USA), DCFH-DA (D6883) and 4-phenylbutyrate (4-PBA, an ERS inhibitor, SML0309, Sigma, St. Louis, USA); DAPI (G1012, Servicebio, Wuhan, China), JC-1 mitochondrial membrane potential detection (MMP) kit (C2006) and ATP detection kit (S2006, Beyotime Biotechnology, Beijing, China), MKT077 (a GRP75 inhibitor, HY15096, MCE, NJ, USA), and GRP75 siRNA (AuGCT Biotechnology, Wuhan, China).

### Cell culture and treatment

Mouse neuroblastoma N2a cells were cultured in 6-well plates or a confocal dish (NEST Biotechnology, China) with 1:1 mixture of DMEM (HyClone) and Opti-MEM (Gibco) containing 10 % fetal bovine serum (FBS; Gibco) in 5 % CO_2_ at 37 °C.

N2a cells were transfected with the pIRES empty vector (pIRES), pEGFP-apoE4 or pEGFP-apoE4 (Δ272–299), or pEGFP-GRP75 plasmid using HighGene Transfection reagent (Abclonal, China), according to the manufacturer’s instruction. At 6 h post transfection, some pEGFP-apoE4 (Δ272–299)-transfected N2a cells were treated with vehicle, 1 mM 4-PBA or 1µΜ MKT077 for 18 h, respectively. Some pEGFP-apoE4 (Δ272–299)-transfected N2a cells were transfected with 50 nM control siRNA or siGRP75 for 24 h. The cells were used for microscopy, quantitative real-time PCR, and Western blot.

### Animals and treatments

Female neuron-specific human apoE4 (Δ272–299) transgenic and age/sex-matched wild-type C57BL/6 mice at 12–14 months old were purchased from Cyagen Company (Cyagen, Guangdong, China). The mice were housed in a specific pathogen-free facility of the Animal Experimental Center of Tongji Medical College with free access to normal chow and water. The mice were injected intraperitoneally with pentobarbital sodium (40 mg/kg) and their brain hippocampal tissues were dissected for subsequent experiments. All experiments were performed in compliance with the guidelines and approved by the Animal Care and Use Committee at the Huazhong University of Science and Technology.

### Western blot

The collected hippocampal tissues were homogenized in RIPA (Tris-HCl, PH 7.5, 50 mM; EDTA, 1 mM; NaCl, 150 mM; 1 % NP-40; 0.1 % SDS; 1 % Triton X-100) with protease inhibitor PMSF and phosphatase inhibitor cocktail. Similarly, the different groups of N2a cells were washed with pre-cold PBS, and lysed in the same lysis buffer. After being centrifuged, the concentrations of proteins were quantified with a BCA Assay Kit. The lysate samples (20–30 µg/lane) were separated on sodium dodecyl sulfate polyacrylamide gel electrophoresis (SDS-PAGE) on 10–12 % gels and transferred onto the PVDF membranes. The membranes were blocked with 5 % skim milk in 0.1 % tween 20 TBST and incubated overnight with primary antibodies overnight at 4 °C. After being washed, the bound antibodies were detected with horseradish peroxidase (HRP)-conjugated secondary antibodies and visualized using the ultrahigh sensitivity ECL Kit in the Bio-Rad Exposure System. The relative levels of target protein to the control β-actin were quantified by densitometric analysis using ImageJ software.

### Mitochondrial morphology analyses

Following treatment, the different groups of N2a cells were incubated with the MitoTracker® Deep Red FM (500 nM) for 30 min at 37℃, washed and visualized under a confocal laser scanning microscope (Nikon, Japan). For morphological quantification of neurites, z-sections were merged (using maximal projection) and the entire lengths (from tip to tip) of mitochondrial neurites that had been labeled with MitoTracker Red were measured. In cell bodies, the mitochondrial lengths were measured in each z-section of the entire cell using ImageJ software. A total of 10 cells were selected randomly from each group and at least 50 mitochondria were measured in each cell.

### ATP measurement

The ATP levels in the different groups of N2a cells were measured using a firefly luciferase-based ATP assay kit, according to the manufacturer’s instructions. Briefly, the different groups of N2a cells were lysed and centrifuged at 12,000 g for 5 min. After determining protein concentrations with a BCA kit, the supernatant samples were mixed with the ATP detection working dilution. The ATP levels were quantified by luminescence and calculated, according to a standard curve established.

### Determination of ROS

The levels of ROS in individual groups of N2a cells were determined using DCFH-DA. Briefly, the different groups of cells were treated with 10 µM DCFH-DA for 20 min at 37°C in the dark. The fluorescent signals in each well of cells were observed under a Nikon Confocal microscope. At least 10 fields of each group of cells were randomly selected, and the green fluorescence intensity of about 100 cells was measured using ImageJ software.

### Measurement of mitochondrial membrane potential (MMP)

The MMP of individual groups of N2a cells was measured using JC-1-based MMP assay kit, according to the manufacturer’s instructions. In brief, the different groups of cells were stained in triplicate with 1 ml of JC-1 staining working solution at 37 °C for 20 min. After being washed, the fluorescent signals were detected using a fluorescence plate reader at 590 nm (red) and 529 nm (green). The ratios of fluorescence intensities at 590 and 529 nm were used to determine the loss of MMP.

### Assessment of co‐localization of ER and mitochondria

The co-localization of the mitochondria and ER in individual groups of N2a cells was analyzed using ER-Tracker™ Blue and MitoTracker® Deep Red FM kits, according to the manufacturer’s protocol. Briefly, the different groups of N2a cells were incubated in triplicate with the fluorescent probes ER-Tracker™ Blue (1 µM) and MitoTracker® Deep Red FM (500 nM) at 37°C for 30 min in the dark. After being washed, the ER-mitochondria co-localization in each well of cells was observed under a laser scanning confocal microscope (Nikon) and analyzed by ImageJ software.

### Measurement of Ca^2+^

The levels of mitochondrial, ER and cytoplasmic Ca^2+^ in individual groups of N2a cells were determined after transfection with mitochondrial, ER and cytoplasmic Ca^2+^ probe plasmids. In brief, N2a cells (5 × 10^4^/dish) were cultured in confocal dishes overnight and transfected in triplicate with pEGFP-apoE4 (Δ272–299) plasmid and three Ca^2+^probe plasmids for 24 h. After being washed, the cells were placed in 1ml of pre-heated Hank’s colorless Ca^2+^ imaging solution and examined under a laser confocal microscope using Nikon confocal imaging software at the time interval of 2 s for 5 min scanning. The baseline Ca^2+^ signal was scanned within 0-60s. At 60s (frame 30), individual wells of cells were stimulated with 1 µM Ionomycin (Sigma) to induce Ca^2+^ release from the ER, and the changes in fluorescent signals of intracellular Ca^2+^ probes were recorded. After scanning, five ROIs were selected randomly in each scan range, and the changes of fluorescent signals of each frame were recorded. The levels of Ca^2+^ in each organelle were quantified using the relative concentration-time variation curve of Ca^2+^ and the baseline fluorescent signal as 1.

### Transmission Electron microscope (TEM)

The impact of apoE4 (Δ272–299) on the mitochondrial morphology and MAM structure in mouse brain tissues was examined by TEM. The dissected hippocampal tissues from individual mice were immersed in 2.5 % glutaraldehyde-2 % paraformaldehyde (4°C) and fixed with 1 % osmic acid-1.5 % potassium ferrocyanide for 1.5 hours. After being washed, the fixed samples were dehydrated and embedded. The ultra-thin tissue sections (50 nm) were double-stained with 3 % uranyl acetate-lead citrate and examined for the mitochondrial morphology and MAM structural changes by TEM.

### Statistical analysis

Data are expressed as means ± S.E.M. The difference among the groups was determined by one-way analysis of variance (ANOVA) and post hoc Fisher’s least significant difference using the SPSS 18.0 statistical software (SPSS, Chicago, IL, USA). The difference was considered statistically significant when a *P-*value *of* < 0.05.

## Results

### ApoE4 (Δ272–299) induces ER Stress ***in vivo*** and ***in vitro***

**Fig. 1:**
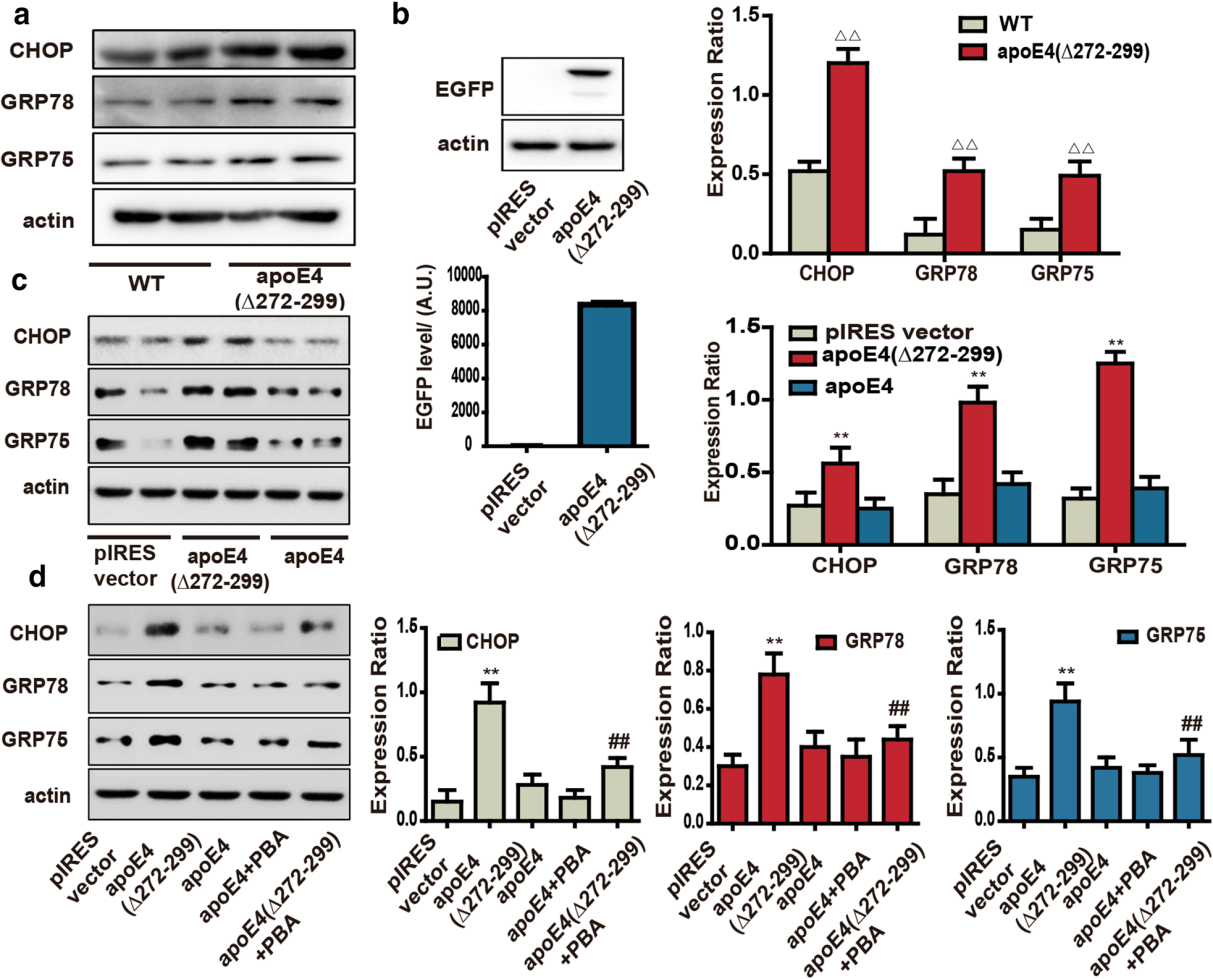
ApoE4 (Δ272–299) triggers ER stress *in vivo* and *in vitro*. **a** Western blot analysis the relative levels of CHOP, GRP78 and GRP75 expression in the hippocampus of apoE4 (Δ272–299) transgenic and the age- and sex-matched wild-type mice. **b** Transfection with pEGFP-apoE4 (Δ272–299) induces EGFP expression in N2a cells. **c** Western blot analysis of the relative levels of CHOP, GRP78 and GRP75 expression in the different groups of N2a cells. **d** Treatment with PBA mitigated the apoE4 (Δ272–299)-up-regulated CHOP, GRP78 and GRP75 expression in N2a cells (^ΔΔ^*P* < 0.01 versus the wild-type mice; **P < 0.01 versus the N2a cells transfected with pIRES vector; ^##^*P* < 0.01 versus the apoE4 (Δ272–299)-expressed cells). Data are representative images or expressed as the mean ± SEM of each group (n = 3) from three separate experiments. The significant differences were determined by ANOVA and post hoc Fisher’s least significant difference

 To determine whether neuronal apoE4 (Δ272–299) expression induces ER stress, we analyzed ER stress-related regulator expression in the hippocampus of neuronal apoE4 (Δ272–299) transgenic and wild-type mice by Western blot assays. First, ApoE4 (Δ272–299) expression was detected in the hippocampus of transgenic mice, but not in wild-type mice (Additional file 1: Figure S1A). As shown in Fig. 1a, the relative levels of CHOP, GRP78 and GRP75 expression in the hippocampus of apoE4 (Δ272–299) transgenic mice were significantly higher than that in the wild-type mice. Immunohistochemistry analysis revealed that GRP78 expression was also higher in the hippocampus and cortex of transgenic mice than that of wild-type mice (Additional file 1: Figure S1B). Similarly, transfection with plasmid for apoE4-(Δ272–299)-EGFP expression induced high levels of EGFP, CHOP, GRP78 and GRP75 expression in N2a cells (Fig. 1b and c). However, induction of apoE4 over-expression did not significantly increase their expression in N2a cells. Immunofluorescence indicated higher levels of GRP78 and GRP75 in the apoE4 (Δ272–299)-EGFP over-expressed N2a cells (Additional file 1: Figure S1C and D). Hence, apoE4 (Δ272–299), but not full length apoE4, expression induced ER stress in neurons. Because PBA can help folding misfolded proteins into correct structure during the process of a classical ER stress, we further tested whether treatment with PBA can mitigate the apoE4 (Δ272–299)-induced ER stress in N2a cells by Western blot. We found that treatment with PBA significantly reduced CHOP, GRP78 and GRP75 expression in the apoE4 (Δ272–299) over-expressed N2a cells (Fig. 1d). Together, such data indicated that apoE4 (Δ272–299) expression induced ER stress in neurons.

### ApoE4 (Δ272–299) changes mitochondrial morphology and function

**Fig. 2:**
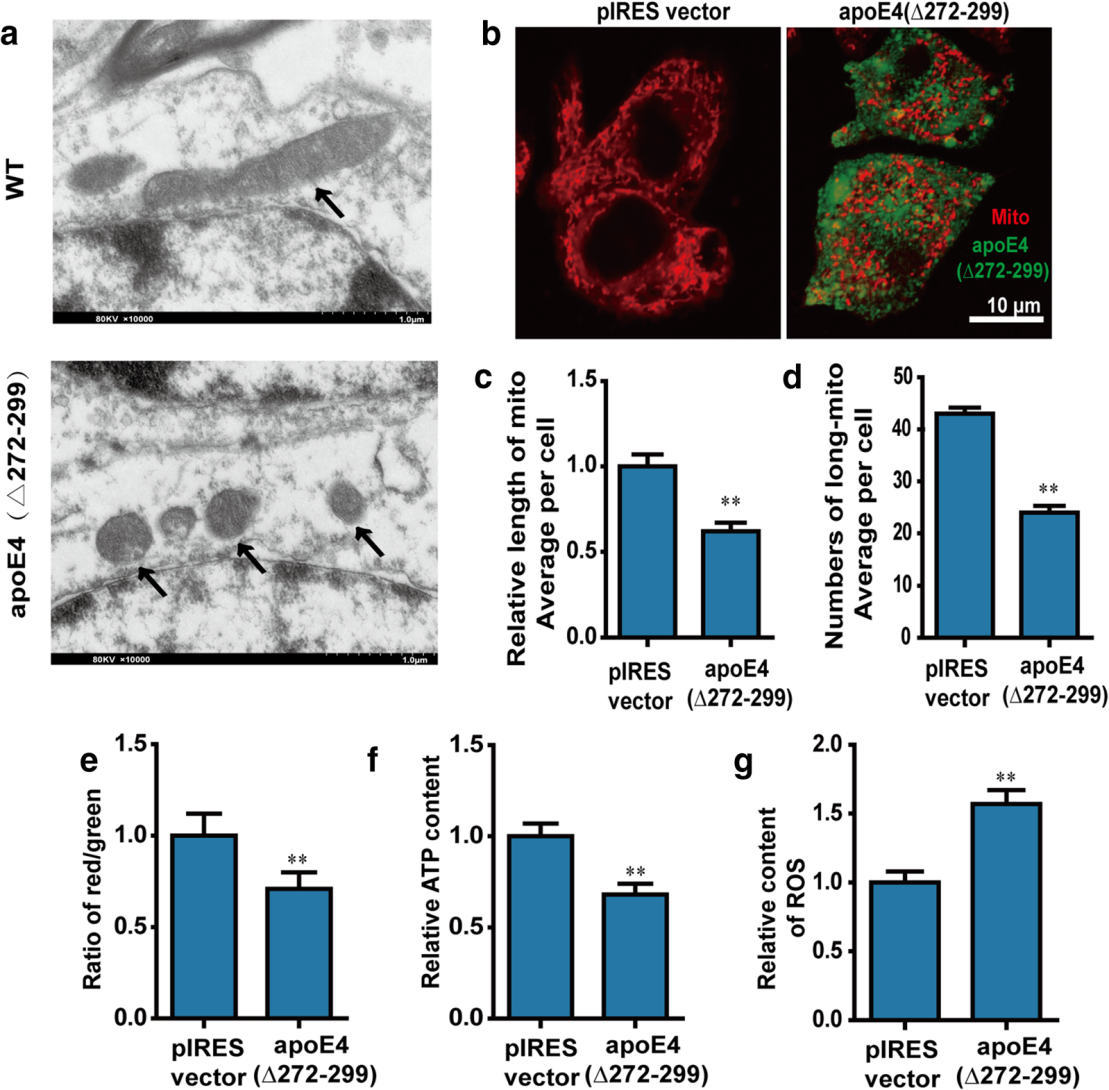
ApoE4 (Δ272–299) induces mitochondrial morphology change and dysfunction. **a** TEM analysis of the morphological changes in mitochondria in the hippocampus of wild-type mice and apoE4 (Δ272–299) transgenic mice. **b** N2a cells were tragnsiently transfected with pIRES or pIRES-apoE4 (Δ272–299) for 24 h, and their mitochondria were stained with mitotracker. The morphological changes in mitochondria were examined under a laser confocal microscope. The scale bar is 10 µm. **c** The length of mitochondria was measured using the ImageJ, n = 20–30 mitochondria per group. **d** The areas of mitochondria was also measured using the ImageJ, n = 20–30 cells. **e** The MMP was detected by the JC-1. **f** The levels of ATP were analyzed by chemiluminescence. **g** The levels of mitochondrial ROS were analyzed by DCFH-DA. Data are representative images or expressed as the mean ± SEM of each group (n = 3) from three separate experiments. **P < 0.01 versus the control N2a cells transfected with pIRES vector, The significant differences were determined by ANOVA and post hoc Fisher’s least significant difference

Because apoE4 (Δ272–299) over-expression also enhanced the expression of GRP75, a critical molecule for the MAM function, we investigated whether apoE4 (Δ272–299) over-expression could affect mitochondrial morphology and function through modulating MAM in neurons. TEM analysis indicated that there were more fragmented and shorter mitochondria with collapsed cristae in the hippocampus of apoE4 (Δ272–299) transgenic mice, relative to that in the wild-type mice (Fig. 2a). Similarly, there were shorter and less number of mitochondria in the apoE4 (Δ272–299) over-expressed N2a cells than that in the control cells (Fig. 2b–d). Hence, apoE4 (Δ272–299) expression caused mitochondrial fragmentation and changed mitochondrial morphology in neurons.

Given that mitochondrial morphology is crucial for its function we examined the impact of apoE4 (Δ272–299) expression on mitochondrial function in N2a cells. The results exhibited that apoE4 (Δ272–299) expression significantly decreased the MMP and ATP production in N2a cells, relative to that in control N2a cells (Fig. 2e and f). In contrast, apoE4 (Δ272–299) expression significantly increased the levels of ROS in N2a cells (Fig. 2g). Thus, apoE4 (Δ272–299) expression disrupted mitochondrial respiratory function and caused oxidative stress in neurons.

### **ApoE4 (Δ272–299) modulates mitochondrial fusion and fission*in vivo*and*in vitro***

**Fig. 3:**
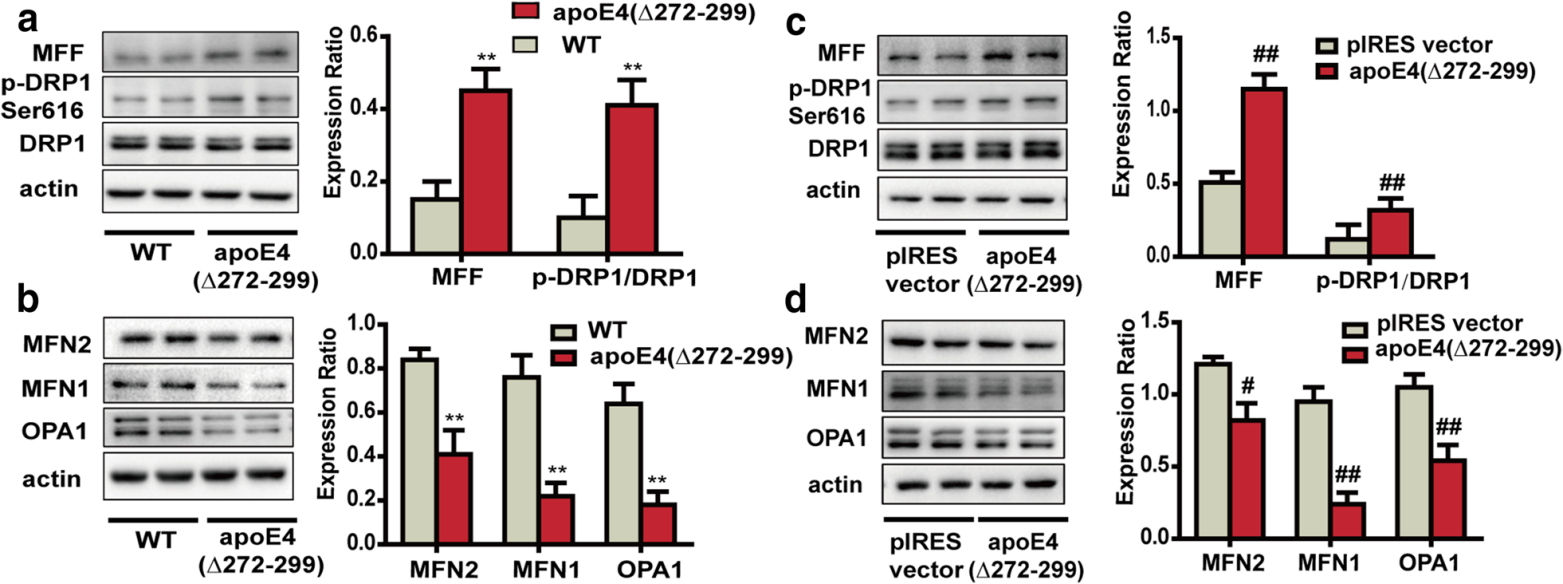
ApoE4 (Δ272–299) induces the imbalance of mitochondrial fusion and fission *in vivo* and *in vitro*. **a**, **b** Western blot analysis of the relative levels of MFF, MFN2, MFN1 and OPA1 expression and DRP1 phosphorylation in the hippocampus of apoE4 (Δ272–299) transgenic and wild-type mice. **c**, **d** Western blot analysis of the relative levels of MFF, MFN2, MFN1 and OPA1 expression and DRP1 phosphorylation in the apoE4 (Δ272–299) expressed N2a and control cells. Data are representative images or expressed as the mean ± SEM of each group (n = 3) from three separate experiments. **P < 0.01 versus the wild-type mice; ^##^*P* < 0.01, ^#^*P* < 0.05 versus the control N2a cells transfected with the pIRES vector. The significant differences were determined by ANOVA and post hoc Fisher’s least significant difference

Next, we investigated the potential mechanism(s) by which apoE4 (Δ272–299) over-expression caused mitochondrial fragmentation. It is well known that mitochondrial morphology is regulated by the dynamic process of mitochondrial fusion and fission, which are dependent on the expression of pro-fusion MFN1/2 and OPA1, and pro-fission MFF and Drp1 proteins. Subsequently, we examined the effect of apoE4 (Δ272–299) over-expression on the relative levels of MFN1/2, OPA1, MFF and Drp1 expression in the hippocampus of mice and in N2a cells by Western blot. In comparison with that in the control mice, the relative levels of MFF expression and Drp1 phosphorylation at Ser616 significantly increased in the hippocampus of apoE4 (Δ272–299) transgenic mice (Fig. 3a), indicating that apoE4 (Δ272–299) promoted pro-fission activity. In contrast, the relative levels of MFN1/2 and OPA1 expression were significantly downregulated in the hippocampus of apoE4 (Δ272–299) transgenic mice (Fig. 3b). Similar patterns of MFN1/2, OPA1, MFF expression and Drp1 phosphorylation were detected in the apoE4 (Δ272–299) over-expressed N2a cells, relative to that in the control cells (Fig. 3c and d). Thus, apoE4 (Δ272–299) over-expression increased pro-fission activity and decreased pro-fusion activity, contributing to mitochondrial fragmentation in neurons.

### Mitochondrial dysfunction induced by apoE4 (Δ272–299) is mitigated by PBA treatment

**Fig. 4:**
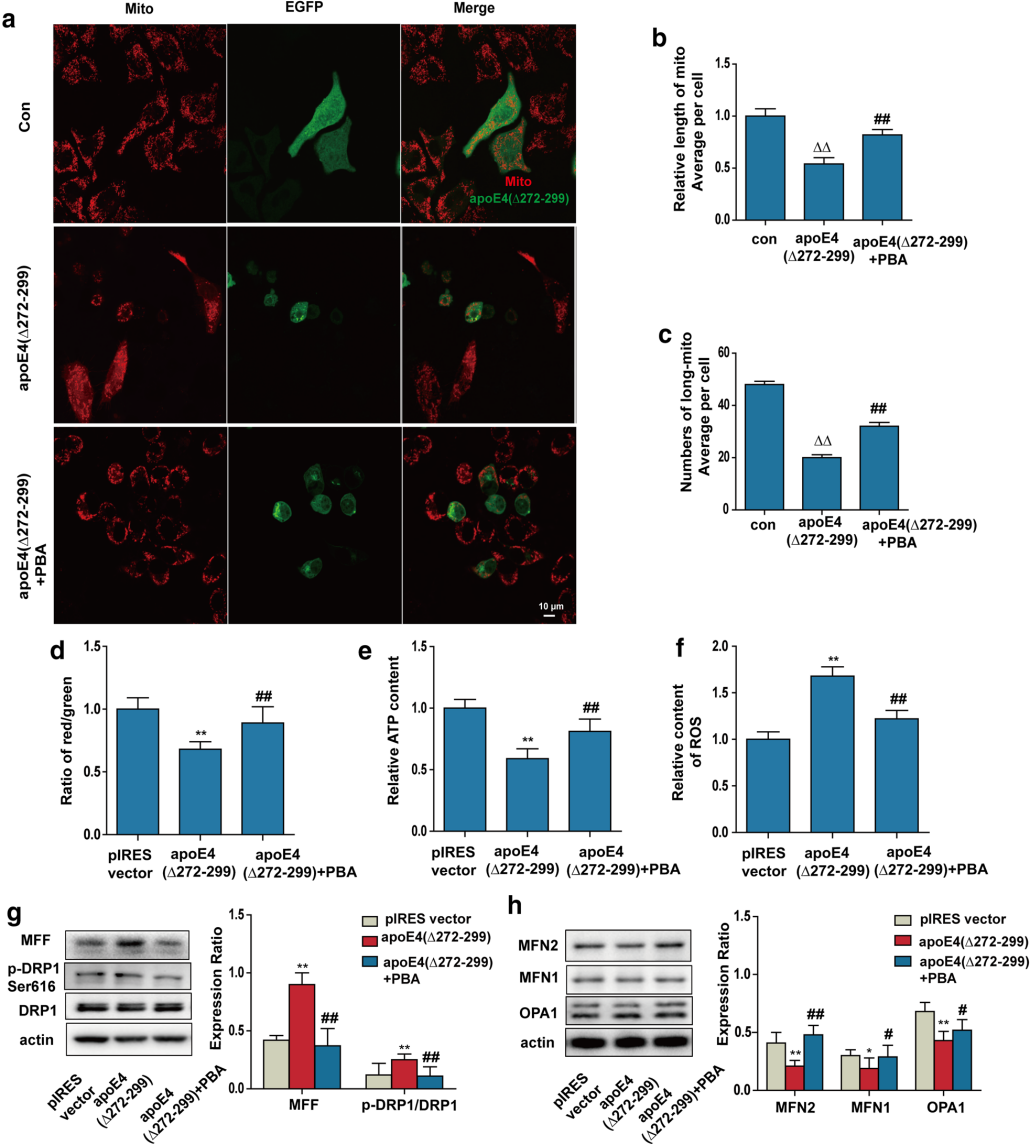
PBA treatment mitigates the apoE4 (272–299)-induced mitochondrial impairment in N2a cells. N2a cells were transiently transfected with apoE4 (Δ272–299), and were treated with, or without, 1 mM PBA for another 18 h. **a** The mitochondria of N2a cells were stained with mitotracker, and were examined by a laser confocal microscope. The scale bar is 10 µm. **b** The lengths of mitochondria were measured using the ImageJ, n = 20–30 mitochondria per group. **c** The areas of mitochondria were measured using the ImageJ, n = 20–30 cells per group. **d** The MMP was analyzed by the JC-1. **e** The levels of ATP were analyzed by chemiluminescence. **f** The levels of ROS were analyzed by DCFH-DA.** g**,** h** Western blot analysis of the relative levels of MFF, MFN2, MFN1 and OPA1 expression and DRP1 phosphorylation in the difference groups of N2a cells. ^ΔΔ^*P* < 0.01 versus the control N2a cells; **P < 0.01,*P < 0.05, versus the vector-transfected N2a cells; ^##^*P* < 0.01, ^#^*P* < 0.05, versus the apoE4 (Δ272–299) expressed N2a cells. Data are representative images or expressed as the mean ± SEM of each group (n = 3) from three separate experiments. The significant differences were determined by ANOVA and post hoc Fisher’s least significant difference

Given that apoE4 (Δ272–299) expression induced ER stress and mitochondrial dysfunction in neurons we tested whether PBA treatment to inhibit ER stress could mitigate the apoE4 (Δ272–299) expression-induced mitochondrial dysfunctions in N2a cells. We found that PBA treatment significantly increased the number and lengths of mitochondria, MMP, ATP levels, but decreased ROS levels in the apoE4 (Δ272–299) expressed N2a cells, relative to that of the PBA-untreated cells (Fig. 4a–f). Thus, PBA treatment to inhibit ER stress rescued the apoE4 (Δ272–299) expression-induced mitochondrial morphological changes and dysfunction in neurons. Further analyses indicated that PBA treatment also decreased the relative levels of MFF expression and Drp1 phosphorylation, but increased MFN1/2 and OPA1 expression in the apoE4 (Δ272–299) expressed N2a cells (Fig. 4g and h). Together, these data further supported that apoE4 (Δ272–299) expression induced ER stress, leading to mitochondrial morphological changes and dysfunction in neurons.

### ApoE4 (Δ272–299) induces MAM coupling via GRP75

**Fig. 5:**
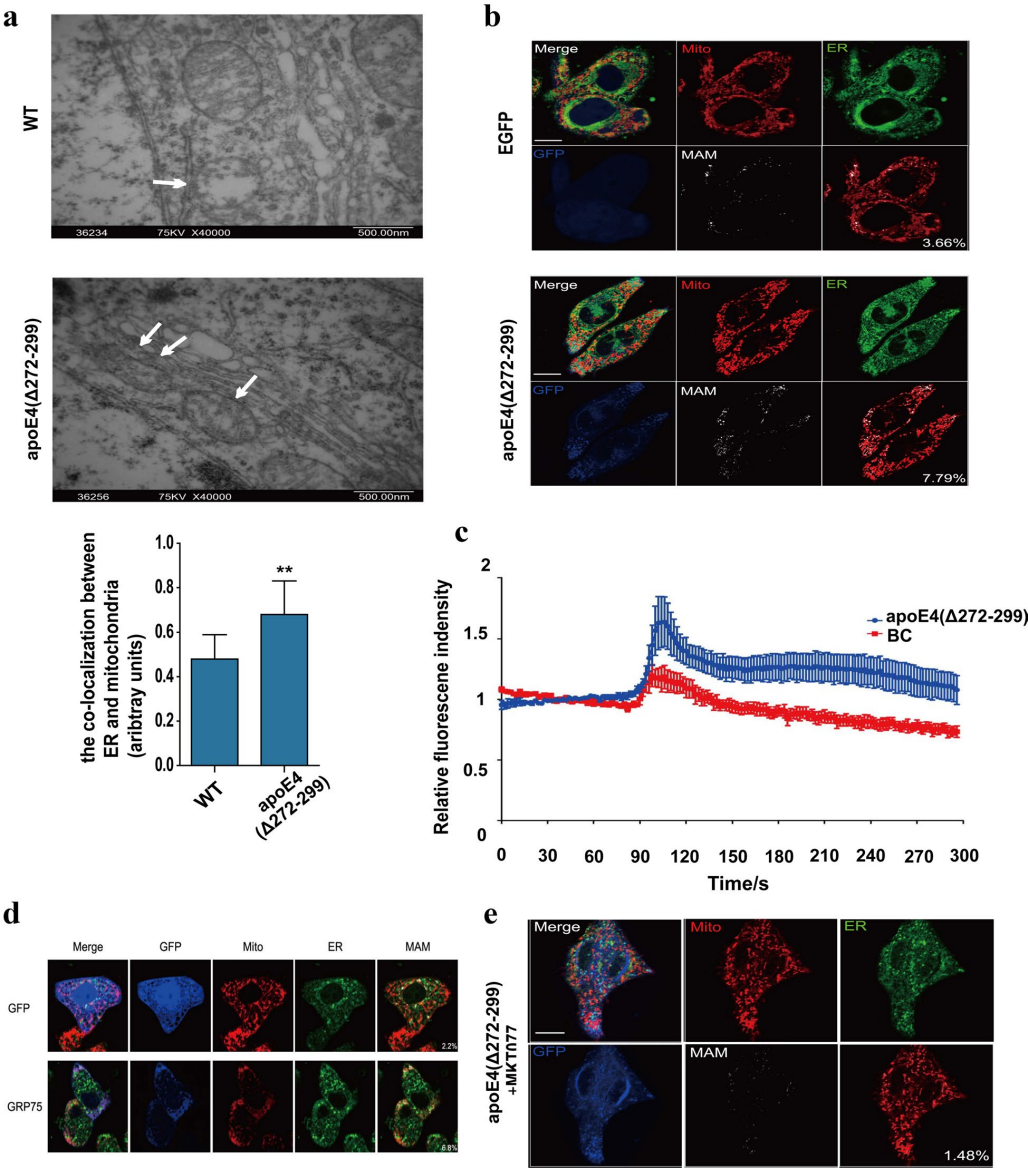
ApoE4 (Δ272–299) promotes ER-mitochondria interaction and mitochondrial Ca^2+^ overload. **a** TEM analysis of the MAM in the hippocampus of wild-type and apoE4 (Δ272–299) transgenic mice. **b** Fluorescent microscopy analysis of MAM in apoE4 (Δ272–299)-expressed N2a cells. The scale bar is 10 µm. **c** Longitudinal analysis of Ca^2+^ levels in mitochondria of N2a cells following inducing apoE4 (Δ272–299) expression. **d** Induction of GRP75 over-expression increased the contact areas of MAM in N2a cells. The scale bar is 10 µm. **e** Treatment with MKT077 mitigated the MAM changes induced by apoE4 (Δ272–299) in N2a cells. The scale bar is 10 µm. Data are representative images or expressed as the mean ± SEM of each group (n = 3) from three separate experiments. **P < 0.01, versus the wild-type mice. The significant differences were determined by ANOVA and post hoc Fisher’s least significant difference

Because GRP75 is an important component of MAM structure, we tested how apoE4 (Δ272–299) expression affected MAM formation. In comparison with the wild-type mice, TEM displayed more mitochondria contacted with ER lumen in the apoE4 (Δ272–299) transgenic mice (Fig. 5a), indicating that apoE4 (Δ272–299) expression induced more MAM formation. Similarly, following transfection with probes for ER and mitochondria, we detected more overlapped areas of mitochondria and ER in the apoE4 (Δ272–299) over-expressed N2a cells, relative to that in the control cells, further supporting that apoE4 (Δ272–299) over-expression induced more MAM formation in N2a cells (Fig. 5b). Because the MAM is crucial for calcium transport, we tested how apoE (Δ272–299) over-expression affected calcium transport into mitochondria in N2a cells. We found that apoE (Δ272–299) over-expression significantly increased calcium transport into mitochondria in N2a cells, relative to that in the control cells (Fig. 5c).

To understand the molecular mechanisms by which apoE4 (Δ272–299) expression enhanced the MAM formation and calcium transport into mitochondria in neurons, we tested how GRP75 regulated the apoE4 (Δ272–299) expression-enhanced MAM formation in N2a cells. We found that induction of GRP75 over-expression significantly increased more MAM formation in N2a cells (Fig. 5d). Furthermore, we observed that the kinetics of GRP75 expression paralleled the formation of MAM (Fig. 5d). In addition, treatment with MKT077, a specific GRP75 inhibitor, significantly inhibited the apoE4 (Δ272–299)-induced MAM formation in N2a cells (Fig. 5e). Collectively, the gain and loss-of-function studies indicated that apoE4 (Δ272–299) induced MAM formation by enhancing GFP75 expression in neurons.

As a “connector”, the GRP75 links VDAC with IP3R to form the calcium channel complex between the ER lumen and mitochondria. We next determined whether apoE4 (Δ272–299) expression would enhance the expression of VDAC and IP3R. Interestingly, apoE4 (Δ272–299) expression did not alter the levels of VDAC and IP3R expression in N2a cells (Additional file 1: Figure S1). Thus, such data indicated that apoE4 (Δ272–299) expression promoted MAM formation by enhancing GRP75 expression in neurons.

### GRP75 is required for apoE4 (Δ272–299)-induced mitochondrial dysfunction

**Fig. 6:**
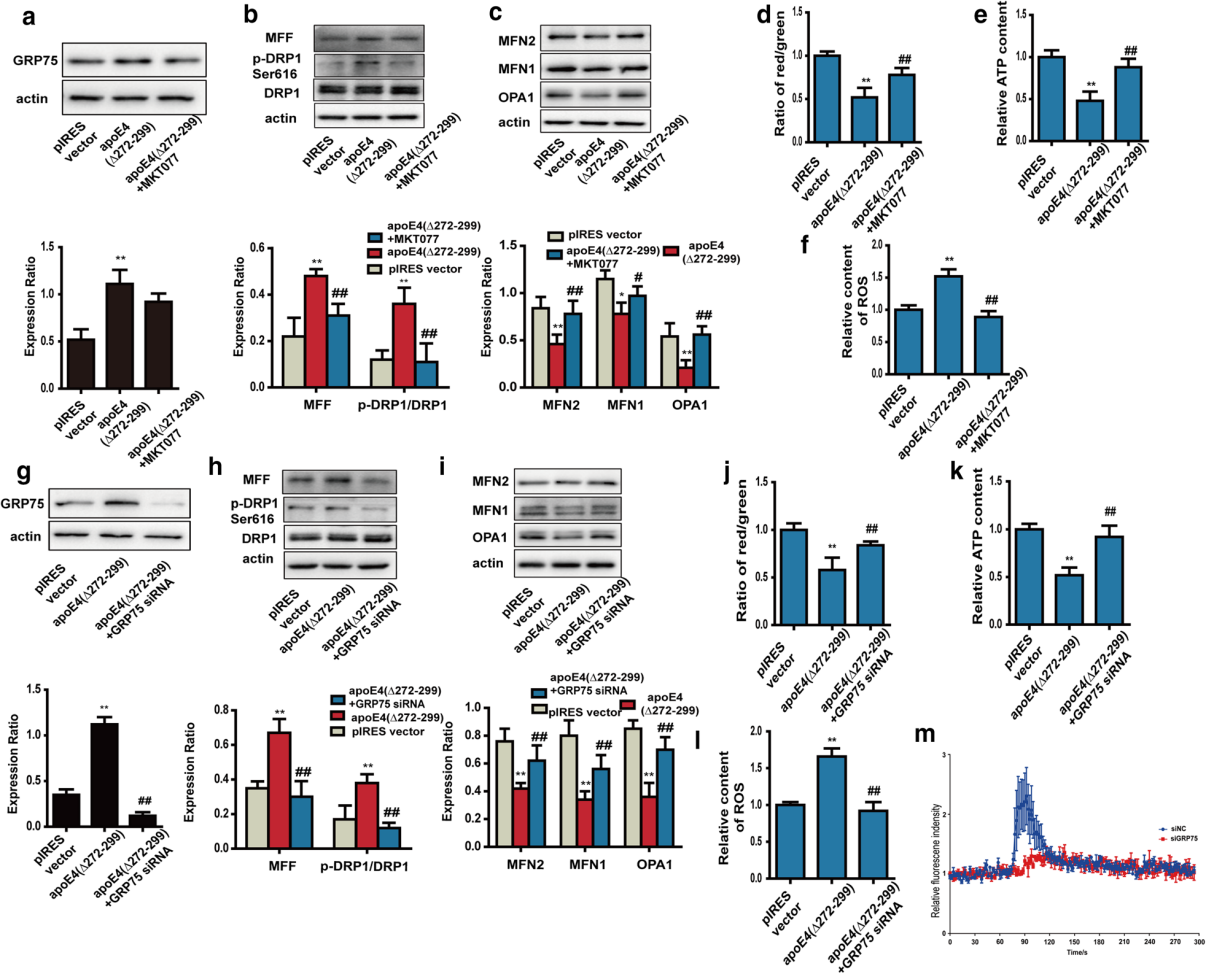
Inhibition of GRP75 alleviates the apoE4 (Δ272–299)-induced mitochondrial impairment in N2a cells. N2a cells were transiently transfected with the plasmid pIRES or apoE4 (Δ272–299), and treated with, or without, 1 µM GRP75 inhibitor MKT077 for 18 h. Furthermore, the apoE4 (Δ272–299)-transfected N2a cells were transfected with 50 nM control or GRP75-specific siRNA for 18 h. **a**, **g** Western blot analysis of the relative levels of GRP75 expression in N2a cells. **b**, **c**, **h**, **i** Western blot analysis of the relative levels of MFF, MFN2, MFN1 and OPA1 expression and DRP1 phosphorylation in N2a cells. **d**, **j** The MMP was analyzed by the JC-1. **e**, **k** The levels of ATP were analyzed by chemiluminescence. **f**, **l** The levels of ROS were analyzed by DCFH-DA. **m** Longitudinal analysis of Ca^2+^ levels in mitochondria of N2a cells. Data are representative images or expressed as the mean ± SEM of each group (n = 3) from three separate experiments. **P < 0.01, *P < 0.05 versus the control N2a cells; ^##^*P* < 0.01, ^#^*P* < 0.05 versus the apoE4 (Δ272–299)-expressed N2a cells. The significant differences were determined by ANOVA and post hoc Fisher’s least significant difference

We further tested that GRP75 was necessary for the apoE4 (Δ272–299)-induced mitochondrial dysfunction in N2a cells. We found that treatment with MKT077 did not significantly reduced GRP75 expression in the apoE4 (Δ272–299) over-expressed N2a cells (Fig. 6a). However, treatment with MKT077 significantly decreased the relative levels of MFF expression and Drp1 phosphorylation, but increased MFN1/2 and OPA1 expression in the apoE4 (Δ272–299) over-expressed N2a cells, relative to that in the untreated control cells (Fig. 6b and c). These indicated that inhibition of GRP75 corrected the imbalance of pro-fission and pro-fusion protein expression induced by apoE4 (Δ272–299) over-expression in N2a cells. Furthermore, treatment with MKT077 also significantly mitigated the apoE4 (Δ272–299) over-expression-decreased MMP and ATP production in the mitochondria and abrogated the apoE4 (Δ272–299) over-expression-increased ROS production in N2a cells (Fig. 6d–f). Similarly, GRP75 silencing by siRNA-based technology also significantly mitigated the imbalance of pro-fission and pro-fusion protein expression (Fig. 6g–i), restored MMP and ATP levels and abrogated an increase in the levels of ROS (Fig. 6j–l) as well as remarkably decreased calcium transport into the mitochondria following ionomycin stimulation (Fig. 6m) in the apoE4 (Δ272–299) over-expressed N2a cells. Collectively, such data demonstrated that apoE4 (Δ272–299) overexpression promoted ER stress and enhanced MAM formation to induce mitochondrial dysfunction by upregulating GRP75 expression, leading to calcium overload in the mitochondria in neurons.

## Discussion

Mitochondrial dysfunction is associated with the development of neurodegeneration diseases, such as AD [29, 30]. In this study, we provided evidence that neuron-specific apoE4 (Δ272–299) expression induced mitochondria dysfunction in vitro and in vivo by triggering ER stress, enhancing MAM formation, leading to mitochondrial calcium overloads. Evidently, we found that apoE4 (Δ272–299) expression significantly decreased ATP production, MMP, but increased ROS production in neurons. The mitochondria dysfunction caused by apoE4 (Δ272–299) expression was likely attributed to up-regulated expression of GRP75, a chaperon protein that is expressed under the circumstance of ER stress and is crucial for the formation of calcium channel IP3R-GRP75-VDAC complex between the ER and mitochondria [31].

ApoE4 is a risk factor for the development of AD [32] and is associated with promoting the formation of amyloid plaques and neurofibrillary tangles [33, 34], neuroinflammation [35], mitochondria dysfunction [36], the blood brain barrier impairment [37] and others. Especially, apoE4-related mitochondrial dysfunction is the key factor for the development of AD. Actually, a recent study reveals that the apoE4 allele alters the expression of many proteins in the mitochondrial function pathway in the brain of mice [38]. Similarly, apoE4 can also change the expression levels of proteins associated with mitochondrial dysfunction in mice under a cerebral ischemic challenge [39]. Thus, apoE4 is a risk factor for mitochondrial dysfunction.

Interestingly, although apoE4 has only two amino acid residues different from apoE3, the apoE4 protein has a more compacted structure and higher potency to form oligomerization than apoE3 [40]. We found that apoE4 (Δ272–299) expression significantly decreased ATP production, MMP, but increased ROS production in neurons. Our findings support the notion that apoE4 may easily harm mitochondrial respiratory chain function and down-regulate respiratory enzyme expression [40]. Because neurons are sensitive to energy supply, the reduced ATP production by apoE4 (Δ272–299) should impair neuronal viability and function [41]. Intriguingly, a previous study has shown that apoE4 can attenuate the neuronal insulin receptor-related signaling by trapping insulin receptor in the endosome, which impairs mitochondrial energy supplement and neuronal function [42]. The impaired insulin receptor signaling and mitochondrial function may form a positive feedback to reduce neuron viability.

In 2001, Huang et al. [43] reported that apoE4 could be cleaved into apoE4 (Δ272–299), which could promote the formation of neurofibrillary tangle-like intracellular inclusion body in neurons of AD patients and N2a cells following inducing apoE4 (Δ272–299) expression [43]. In this study, we observed filament like apoE4 (Δ272–299)-GFP signals in transfected N2a cells. This, together with the fact of apoE4 (Δ272–299) inducing ER stress in neurons, suggests that apoE4 (Δ272–299) may be unable to fold properly into a correct structure to form oligomerization, leading to ER stress in neurons.

The ER stress is an important player in the development of AD and other neurodegeneration diseases [44]. Our previous study has shown that apoE4 (Δ272–299) can trigger ER stress and induce tau hyperphosphorylation and axonopathy in neurons [45]. In this study, we revealed that the ER stress induced by apoE4 (Δ272–299) promoted mitochondrial dysfunction and mitochondrial calcium overload, which should contribute to the neurotoxicity of apoE4 (Δ272–299). Mechanistically, we found that the ER stress induced by apoE4 (Δ272–299) significantly upregulated the expression of GRP75 both *in vivo* and *in vitro*, which were abrogated by treatment with PBA. It is possible that the enhanced ER stress may up-regulate the expression and activity of transcription factors, such as ATF4, CHOP and others that promote the expression of GRP75. We are interested in further investigating which transcription factor up-regulates GRP75 expression in neurons under an ER stress condition.

In this study, we found that apoE4 (Δ272–299) expression significantly promoted the MAM formation and mitochondrial calcium loads in neurons in a GRP75-dependent manner. Evidently, GRP75 over-expression significantly induced the formation of MAM and inhibition of GRP75 activity by treatment with MKT077 abrogated the apoE4 (Δ272–299) over-expression-induced MAM formation and mitochondrial calcium overload in neurons. These findings indicated that apoE4 (Δ272–299) expression enhanced the MAM activity and extended previous observations in astrocytes by disrupting the lipid synthesis function of MAM [46]. Thus, our findings suggest that the ER stress and GRP75 may be new therapeutic targets for intervention of AD.

The MAM is crucial for mitochondrial function to regulate cholesterol metabolism [47], autophagy [48], intracellular calcium homeostasis [49] and others. There are several molecular models to describe the calcium transport channel between the ER and mitochondria, including the IP3R1-GRP75-VDAC1 complex [31]. Calcium can be transported from the ER through the complex channels into the mitochondria to maintain mitochondrial calcium homeostasis. Conceivably, increased MAM formation and activity should promote mitochondrial calcium overload, which is associated with the pathogenesis of various diseases, such as stroke [50], acute myocardium ischemia [51]. Mitochondrial calcium overload may cause respiratory outbreak and strikingly enhance the production of ROS, leading to mitochondrial dysfunction [52]. We observed that apoE4 (Δ272–299) expression decreased MMP and ATP production, but increased ROS production in N2a cells, the hallmarks of mitochondrial dysfunction. Interestingly, we found that apoE4 (Δ272–299) expression did not significantly alter the levels of IP3R1 and VDAC1 expression between the apoE4 (Δ272–299) expressed and control N2a cells. Furthermore, inhibition of GRP75 completely abrogated the apoE4 (Δ272–299)-induced mitochondria dysfunction. Hence, the up-regulated GRP75 expression by apoE4 (Δ272–299) is crucial for the increased MAM formation and mitochondrial calcium load, leading to mitochondrial dysfunction in neurons. Such novel findings may shed new lights in molecular mechanisms underlying the action of apoE4 (Δ272–299) in inducing mitochondrial dysfunction and neurotoxicity.

Mitochondrial morphology and dynamics are critical for mitochondrial function [53]. Mitochondrial morphology is regulated by the dynamic process of mitochondrial fission and fusion, which are dependent on the relative levels of MFF expression and Drp1 phosphorylation and Mfn1/2 and OPA1 expression, respectively [54]. We found that apoE4 (Δ272–299) over-expression significantly decreased Mfn1/2 and OPA1 expression, but increased MFF expression and Drp1 phosphorylation in neurons, indicating that apoE4 (Δ272–299) promoted mitochondrial fission, but inhibited mitochondrial fusion in neuron. Consequently, we detected mitochondrial fragmentation and other morphological changes in the hippocampus of apoE4 (Δ272–299) transgenic mice and apoE4 (Δ272–299) expressed N2a cells. Importantly, inhibition of GRP75 expression and activity abrogated the apoE4 (Δ272–299)-changed mitochondrial dynamics and morphology in neurons. The morphological changes induced by apoE4 (Δ272–299) may be the basis of mitochondrial dysfunction in neurons. We are interested in further investigating how GRP75 regulates the mitochondrial dynamics in neurons.

In conclusion, our data revealed that apoE4 (Δ272–299), a specific N-terminal fragment of apoE4, significantly impaired neuron mitochondrial function both *in vivo* and *in vitro* by triggering ER stress, up-regulating GRP75 and subsequently increasing MAM formation, leading to mitochondrial calcium overload. Inhibition of GRP75 expression by siRNA or activity by MKT077 mitigated or abrogated the apoE4 (Δ272–299) expression-induced mitochondrial dysfunction in neurons. Hence, GRP75 may be a potential therapeutic target for ameliorating the neurotoxicity of apoE4 (Δ272–299), and our findings may uncover the molecular mechanisms by which GRP75 regulates the MAM calcium channel, contributing to the pathogenesis of neurodegenerative diseases.

## Supplementary Information

**Additional file 1: Figure S1.** ApoE4 (Δ272–299) over-expression triggers ER stress in vivo and in vitro. (A) Western blot analysis of apoE4 (Δ272–299) expression in the hippocampus of transgenic and wild-type mice. (B) Representative immunohistochemistry images of GRP78 expression in the hippocampus and cortex of transgenic or wild type mice. (C) Representative immunofluorescent images of GRP78 expression in the control and apoE4 (Δ272–299)-EGFP over-expressed N2a cells. (D) Representative immunofluorescent images of GRP75 expression in the control and apoE4 (Δ272–299)-EGFP over-expressed N2a cells.

## Data Availability

All the data or materials involved in the research are available if required.
