# Supplementary figures and images for: ApoE4 (Δ272–299) induces mitochondrial‐associated membrane formation and mitochondrial impairment by enhancing GRP75-modulated mitochondrial calcium overload in neuron
Source: Cell Biosci. 2021 Mar 6;11:50. doi: 10.1186/s13578-021-00563-y (PMC7937300; doi:10.1186/s13578-021-00563-y)

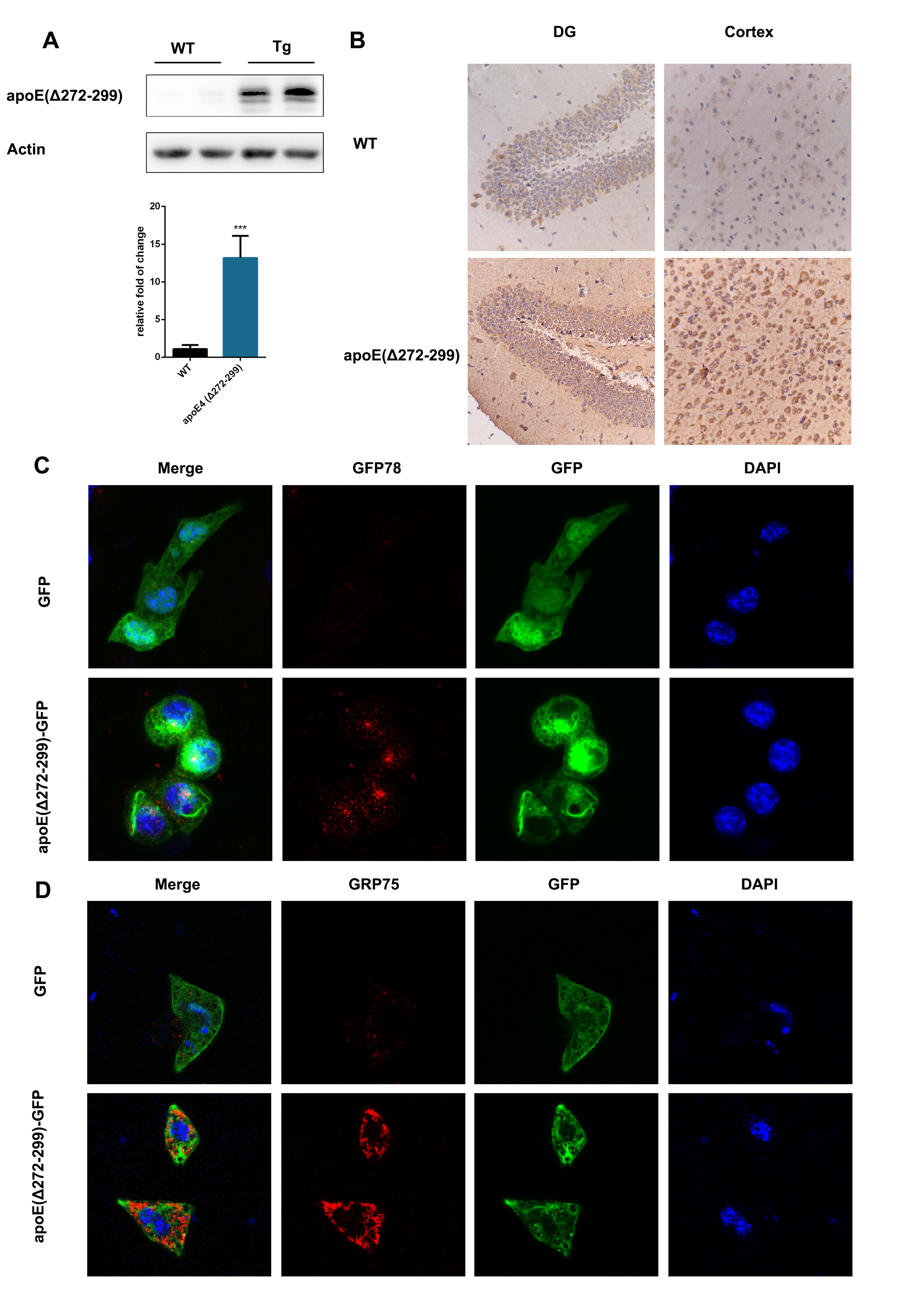

Supplement: Supplementary file 1 — Additional file 1: Figure S1. ApoE4 (Δ272–299) over-expression triggers ER stress in vivo and in vitro. (A) Western blot analysis of apoE4 (Δ272–299) expression in the hippocampus of transgenic and wild-type mice. (B) Representative immunohistochemistry images of GRP78 expression in the hippocampus and cortex of transgenic or wild type mice. (C) Representative immunofluorescent images of GRP78 expression in the control and apoE4 (Δ272–299)-EGFP over-expressed N2a cells. (D) Representative immunofluorescent images of GRP75 expression in the control and apoE4 (Δ272–299)-EGFP over-expressed N2a cells. [file 13578_2021_563_MOESM1_ESM.tif]
